# Does employee engagement promote innovation? The Facilitators of innovative workplace behavior via mediation and moderation

**DOI:** 10.1016/j.heliyon.2023.e21817

**Published:** 2023-11-08

**Authors:** Bilqees Ghani, Syed Irfan Hyder, Sunghoon Yoo, Heesup Han

**Affiliations:** a Institute of Business Management, HRM and Management Department, Karachi, Pakistan; b Ziauddin University, Karachi, Pakistan; c Audit Team, Hanmoo Convention (Oakwood Premier), 49, Teheran-ro 87-gil, Gangnam-gu, Seoul, 06164, South Korea; d College of Hospitality and Tourism Management, Sejong University, 98 Gunja-Dong, Gwanjin-Gu, Seoul 143-747 South Korea

**Keywords:** Employee engagement, Employee voice behavior, Innovative work behavior, Perceived distributive fairness

## Abstract

The demonstration of innovative behaviour by employees plays a crucial role in enabling organisations to effectively respond and adapt to the rapidly evolving business landscape. There has been an increase in research aimed at identifying the factors that contribute to the progressive development of innovative capacity, as there has been a substantial rise in interest in the comprehension of innovation mechanisms. Drawing on self-determination theory, this study aims to investigate the effect of employee engagement on innovative work behavior. The mediating role of employee voice behavior was also explored. Additionally, this study examines the potential moderating impact of perceived distributive justice on the relationship between employee engagement and innovative work behavior. A quantitative study was undertaken using a sample size of 180 participants who were employed in the manufacturing sector of Pakistan. Partial least squares structural equation modelling (PLS-SEM) was used to analyze the results of the study. The results revealed that engaged employees are more likely to exhibit innovative behavior. Furthermore, employee voice was discovered to fully mediate the relationship between employee engagement and innovative behavior, as well as to have a significant influence on both employee engagement and innovative behavior. However, the findings did not support the hypothesis that perceived distributive justice moderates the association between employee engagement and innovative behavior. Future research directions and managerial implications were also mentioned.

## 1 Introduction

Manufacturers are vital to Pakistan's economic growth and success. It creates jobs, transfers foreign cash, and drives industrial growth [1]. Significantly, approximately 12.79% of Pakistan's GDP growth can be attributed to this sector. Industrialization affects society as well as the economy. It combats unemployment by producing jobs for all skill levels, reducing poverty, and promoting socioeconomic stability [2,3]. Research shows that creativity and innovation are crucial to success in today's economy and manufacturing industry, given Pakistan's manufacturing sector's importance to economic growth. Creativity plants the seeds of new products, services, solutions, and procedures [4]; innovation flourishes and blossoms when a company tends to the seeds with tender, loving care [5]. There are continuing efforts to operationalize creative thoughts that could lead to innovation. Innovation is long-term and involves progress, setbacks, and other developments [6]. It demands mental, emotional, and physical work from the individual and specific conditions. According to studies, innovative behavior among employees is crucial for the idea generation process of innovation, as it helps in the transition to more efficient innovation, whether in the product line or in the generation of new ideas [7,8]. Because creativity and innovative behaviors are commonly seen as essential components of preserving competitiveness in this age of globalization, this research focuses on these ideas. By examining the significance of innovative work behavior in the manufacturing context, previous research has identified a number of factors that can predict employee innovative work behavior [9,10,11,12,13]. However, employee engagement, a key factor in innovative work behavior, has been ignored. Without engagement, employees won't understand the company's plan, lose focus, become disoriented, and move in opposite directions, resulting in undesirable outcomes [14]. Engaged employees feel ownership and purpose in their jobs, which inspires them to take initiative and help the company succeed. They experiment and take risks because their managers and colleagues empower and support them. If a company fails to engage its staff, they may lose confidence and be unable to innovate [15]. This study thus reveals a direct link between employee engagement and innovative work behavior in Pakistan's manufacturing setting, which is the primary goal of this study.

Several studies have linked workplace attitudes to employee performance. Employee engagement is one of the few attitudes recognized as a significant predictor of employee success, in addition to job satisfaction, job participation, etc. [16]. Engaged employees work smarter and more successfully, and employee engagement supports desired behavior, including innovation, according to Ref. [17]. Although previous research strongly supports the link between employee engagement and innovative behavior, some studies have found the relationship ambiguous, so a mediator is needed to clearly explain the relationship between EE and IWB [18]. We suggest that employee voice behavior can mediate this relationship. Employee voice refers to how much employees are encouraged and enabled to openly communicate their thoughts, grievances, and suggestions in the workplace. When employees enjoy their work and feel like they're making a difference, they're more likely to speak up and recommend organizational improvements. Employee engagement encourages innovative work behaviour, and employee voice links this engagement to practical ideas and innovative activities [19]. Voice behavior as a mediator between EE and IWB is another study objective.

It was anticipated that innovative behavior is influenced not only by innate traits such as employee engagement but also by the combined effect of organizational structures, which magnify its impact [20]. We propose that perceived distributive justice moderates the association between employee engagement and innovation. Employees' perceptions of fairness and equity in how awards, praise, and resources are divided within the organization are referred to as perceived distributive justice. The perceptions of fairness among engaged individuals have a positive influence on their level of engagement, resulting in sustained levels of innovation and organizational performance. Thus, the third key objective of this study is to analyze how perceived distributive fairness moderates’ employee engagement and innovative behavior.

Overall, the purpose of this study is to contribute to the behavioural literature, particularly in the context of manufacturing, in the three significant ways described above. This study assists policymakers, human resource managers, and manufacturing industry stakeholders in promoting innovative employee behaviour.

## 2 Literature review

A literature review on the impact of employee engagement on employees' innovative behavior through the mediation of employee voice and moderation of perceived distributive fairness provides an overview of the current state of research on the relationship between these variables. This review considers studies from various disciplines, including psychology, management, and organizational behavior, to examine the role that employee engagement plays in promoting employees' innovative behavior and how it is affected by employee voice and perceived distributive fairness. By synthesizing the findings of these studies, this review aims to provide a comprehensive understanding of the interplay between these variables and their impact on employee behavior. Ultimately, the goal of this literature review is to offer insights into the importance of promoting employee engagement as a means to encourage innovation and foster a positive work environment.

### 2.1 Employee engagement

Employee engagement is defined as a state of mind characterised by vigour, commitment, and absorption in regard to an individual's effort/work [21]. This positive attitude in the workplace may be communicated as strong engagement, which is the opposite of fatigue. When a company's whole staff is committed to achieving its objectives, it has attained full employee engagement [22]. Employee engagement can be classified into three distinct types: emotional, cognitive, and behavioural. Employees who exhibit emotional engagement demonstrate positive affective states towards their work and the business, including but not limited to enthusiasm, pride, and passion. The level of focus and concentration that employees have on their jobs, implying a profound immersion in their work, is referred to as cognitive engagement. Proactive habits like going the extra mile, taking the initiative, and actively participating in problem-solving and decision-making processes demonstrate behavioural engagement [23]. A combination of various forms of engagement produces a workforce that is committed to achieving organisational objectives as well as invested in the expansion and success of the business as a whole.

### 2.2 Innovative behavior

In research investigations, innovation and creativity are often used interchangeably [24]. Innovation is the development, adoption, and implementation of new ideas [25], while creativity is merely the invention of new ideas [26,27]. Innovative Behavior is the purposeful act of creating, implementing, and proposing novel ideas in order to improve the performance of a person, department, or organization [24]. Particularly, the innovative behaviour of workers is a valuable asset since it entails the creation, adoption, and implementation of novel ideas for labor, goods, and services, which helps any organization prosper in an innovative business environment [24]. Individuals benefit from innovative behavior because it emphasizes individual roles rather than those of the institution. Innovative Behavior also includes activities that may be adopted by other new ideas for the company, indicating that this form of behaviour is essential as a source of sustained competitive advantage for businesses [28,4].

### 2.3 Employee voice

Innovative employee behavior at work results in an innovative business and fosters a pattern of bolstering employee voice [29]. Employee voice is a component of an employee's engagement at his or her company. According to Ref. [30], employee voice is a catalyst for appreciating an organization's innovative know-how, boosting firm performance [31], gaining and retaining pleased consumers, and gaining and retaining satisfied employees [32]. Employee voice is much more than just a metric of improved employee reputation [33]. It is crucial to pay attention to employee voice since it contains innovative ideas, proposals, and opinions. According to Ref. [34], innovation can only be realized through employee creativity; hence, the employee voice must be heard and considered. According to studies, employees who fear receiving negative feedback from their managers tend to suppress their voice and choose to remain silent [35]. As a result, companies should now adopt a 360-degree innovation approach in which employee ideas and suggestions should be heard and acknowledged from all angles, as encouraging employee voice at the workplace will motivate employees to demonstrate a high level of innovative workplace behaviour [36]. Employees exhibit zeal for their jobs when they think they are being heard and their suggestions are being considered [37]. Increased employee engagement occurs when employees' thoughts, ideas, and perspectives are heard, making them feel valued and integral to the firm. Employee input is a motivational aspect that supports creative idea generation [38].

### 2.4 Perceived distributive fairness

Perceived distributive fairness is distributive fairness, which refers to the distribution of benefits and expenses among workgroups. For instance, when there is an uneven distribution of compensation, i.e., when all workers get the same pay or salary despite the fact that a few have worked longer hours and outperformed others, this is an example of pay inequality [39]. Perceived distributive fairness is the view that all workers have at work about whether or not they are receiving compensation for their labour [12]. The expectations of employees are connected to their notion of perceived distributional justice [40]. When their view of work and reward is balanced, employees are more receptive to innovation. There is a relationship between perceived distributive justice and job engagement, and this relationship leads to workers modifying their attitudes and actions, which increases employee innovation in the workplace [41]. Therefore, perceived distributive justice may moderate the relationship between employee engagement and creative activity.

### 2.5 Theoretical background and hypothesis development

#### 2.5.1 Self-determination theory

The Self-Determination Theory (SDT) provides a solid theoretical framework for understanding the intricate relationship between employee engagement and innovative work behaviour [42]. This framework addresses the influence of employee voice behavior as a mediating element and the role of perceived distributive fairness as a moderating component. Self-Determination Theory (SDT) emphasizes the inherent human need for autonomy, competence, and relatedness in the context of the job. Employees are more likely to engage in task engagement and develop new ideas when they feel a feeling of autonomy and control over their job, have the required skills for good performance, and work in a positive and supportive atmosphere [43].

Moreover, the (SDT) theoretical framework provides support for the notion that employee voice behaviour plays a significant role in this dynamic context. Employees are empowered and encouraged to actively participate in enhancing the organisational success by being given a safe environment to express their ideas, concerns, and suggestions without fear of repercussions when there is an open communication culture in place [44]. It has been discovered that the presence of an environment in which individuals perceive their ideas to be acknowledged and incorporated into the decision-making process increases their intrinsic motivation and fosters a greater commitment to engaging in innovative work behaviours.

In addition to this, employees are more likely to have better social connections and a stronger sense of belonging in an organisation where justice prevails along with feeling of positive emotional state and a greater level of engagement [45]. This results in searching out new ideas, collaborating with coworkers, and providing insightful feedback to improve the organization's processes and products.

#### 2.5.2 The relationship between employee engagement and innovative behavior

Previous studies have shown that individuals who are more involved in their work demonstrate a higher level of creativity. Resilience and engaged behaviors such as persistence may aid individuals in overcoming obstacles [46]. According to Ref. [47], there is a strong connection between job fulfilment (i.e., employee engagement) and task innovation. In particular, research on the topic of intrinsic motivation reveals a connection between actually engaging in one's work and an innovative nature [48,49]. According to Ref. [50], workers who are engaged in their work are more likely to exhibit innovative behavior. It has been found that innovative behavior and work engagement have a close connection that is beneficial, and that psychological aspects (engagement) have no detrimental influence on innovative behavior. This is a very intriguing finding.

H1 Employee engagement has a positive and significant effect on innovative behavior.

#### 2.5.3 Mediating of employee voice between the relationship of employee engagement and innovative behavior

Social Determination Theory (SDT) provides a comprehensive framework for understanding the intricate relationship between employee voice behaviour, employee engagement, and innovative work behaviour within organizational contexts [51]. Research indicates that engaged employees play a crucial role in promoting autonomy, competence, and relatedness within the workforce [52]. Supported by self-determination theory, this heightened level of participation cultivates a conducive atmosphere wherein employees are empowered and motivated to openly articulate their viewpoints, recommendations, and ideas [53]. This mediation function of employee voice behaviour emerges as a critical link through which the favorable influence of employee engagement transfers into enhanced innovative work behaviour [54]. As engaged employees offer their unique perspectives by sharing their thoughts, firms are more likely to capitalize on this abundance of ideas, promoting a culture of collaborative creativity [55]. Moreover, research by Ref. [56] shows that engaged employees are more likely to offer creative and useful ideas [36]. Their input on work-related concerns handled with the purpose of strengthening the organization is one component that contributes to enhanced organizational creativity and, as a result, innovative behavior [38]. Therefore, the examination of employee voice behaviour as a mediator between employee engagement and innovative work behaviour indicates the interconnectedness of these concepts, emphasizing the significance of transparent communication and inclusive decision-making in fostering organizational innovation and advancement [57]. We, therefore, formulate the following hypothesis.

H2 Employee Voice has a mediating role between the relationship of employee engagement and innovative work behavior.

#### 2.5.4 Moderation of perceived distributive fairness between the relationship of employee engagement and innovative behavior

Perceived distributive justice incorporates an individual's subjective evaluation or perception of the equitable and fair distribution of resources, rewards, opportunities, or burdens within the framework of a society, organization, or any given social setting. It concentrates on how individuals perceive and assess the distribution of these resources in relation to their sense of what is just and fair [58]. We propose that, within the context of Self-Determination Theory (SDT), perceived distributive fairness can serve as a moderator in the relationship between employee engagement and innovative work behaviour. According to SDT, individuals are intrinsically driven when their basic psychological needs for autonomy, competence, and relatedness are addressed. Employee engagement reflects this inherent desire, since engaged people easily involve themselves in their work [59]. As a contextual component, perceived distributive justice determines the nature of this engagement [60]. When these two aspects interact, such as employees' perceptions of fairness in the distribution of rewards and resources and their underlying psychological needs, i.e. engagement, they are more likely to generate an environment conducive to innovative work behaviour [61]. However, the perception of distributive injustice can hinder this process. Individual intrinsic drive is inhibited, and the beneficial relationship between engagement and innovation is hampered [62]. Therefore, in the context of SDT, perceived distributive justice serves as a crucial moderator, determining the extent to which engaged employees can channel their intrinsic motivation into innovative endeavors, thereby steering an organization towards greater creativity and competitiveness. We thus propose the following hypothesis.

H3 The relationship between Employee engagement & Innovative behavior would strengthen if the value of perceived distributive fairness would be high & weaken if the value of perceived distributive fairness would be low.

**Fig. 1:**
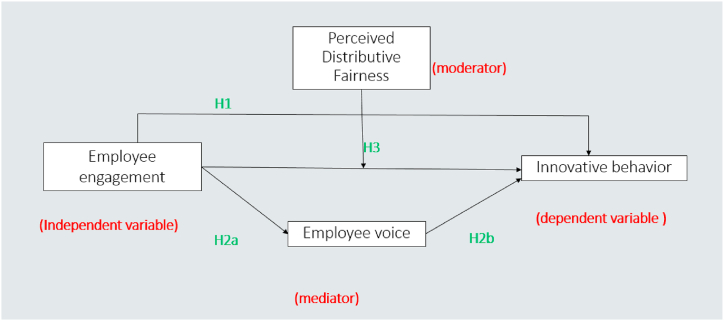
Conceptual framework.

Based on above hypotheses, a conceptual framework/model has been developed depicted as Fig. 1.

## 3 Methodology

### 3.1 Participants

The study employed a quantitative methodology, specifically a causal-explanatory research design, to examine the cause-and-effect relationship between employee engagement and innovative behaviour within the context of Pakistan's manufacturing industry. The investigation focused on Pakistan's manufacturing sector, a significant contributor to the country's gross domestic product, exports, and employment. This emphasis provides a valuable opportunity to identify avenues for enhancing employee engagement, fostering innovation, and ultimately enhancing industrial performance. To confirm the validity of the study's findings, data was obtained from a carefully selected sample of 180 people using convenience sampling. This sample size was calculated to achieve an optimal equilibrium between statistical power and precision of results, taking into account both effect size and level of significance. The sample-to-item ratio was used to determine the sample size for this study [63]. The sample size can be calculated by multiplying the total number of items, given as 21, by a factor of 10, yielding a sample size of 210, with a sample size of 180 falling within this range. Furthermore [64], recommend a sample size of 100 or greater. This confirms our sample size of 180 even more. The procedure for collecting data commenced by identifying participants working in Pakistan's manufacturing sector, who were then provided with the self-administered questionnaire via email and in-person interactions. Respondents were assured of the confidentiality and anonymity of their responses, and they were given clear instructions to ensure the accuracy and consistency of their responses. This methodical approach demonstrates the study's dedication to documenting comprehensive and trustworthy insights into the relationship between employee engagement and innovative behaviour in Pakistan's industrial landscape.

To address the common method variance (CMV), Marker variable approach [65] was utilised to reduce the risk of common method bias due to the data's sole source. This strategy suggests including variables that are theoretically unrelated in the data capture instrument in order to obtain unbiased responses [65]. propose that the marker variable scale should be placed immediately after the theoretically pertinent constructs and before the dependent variable. In addition, the construct should contain at least four items in order to reduce common method variance by 70 % [66]. Moreover, to reduce non-response bias and verify our study's dataset's representativeness, we performed rigorous data imputation. Non-response bias in survey research can lead to insufficient data and invalid findings. This bias was reduced by using data imputation to anticipate missing values for non-respondents based on participant characteristics and responses. This strategy allowed us to have a more comprehensive and well-balanced dataset, thereby reducing the likelihood of bias due to missing data [67].

### 3.2 Measurements/scales

In [68] developed a seven-item scale for measuring employee engagement. One of the sample items on this scale was "I find my work to be meaningful and purposeful." Similarly, employee voice behaviour was evaluated using a 5-item scale developed by Ref. [69]. Among the components of this scale, "I easily obtain opportunities to express my views upward" was one example. In Ref. [70] was used to assess the perception of distributive justice. "The rewards I receive are not proportional to my investments" was one of the examples provided. Finally [68], developed a four-item measure to assess innovative work behaviour. Example item for this scale was "I easily generate creative ideas." These meticulously selected scales provide a comprehensive method for measuring employee engagement, voice behaviour, perceptions of distributive justice, and innovative work behaviour.

### 3.3 Statistical method

This study employed PLS-Validity, SEM reliability, mediation, and moderating analyses to evaluate the hypotheses presented in this research report on innovative employee behavior in the context of employee engagement. The study aimed to determine the relationship between employee engagement and innovative behavior, with employee voice as a mediator between innovative behavior and employee engagement, and perceived distributive justice as a moderator.

Table 1 shows the cross-tabulation between education and gender, and it demonstrates that men are more interested in obtaining various educational degrees. The analysis shows that males are more likely to complete their O-levels than females. The same goes for A-level. For bachelor's degrees, the largest number of females is 46. In contrast to O'levels or matriculation, M.Phil. carries one point and Ph.D. carries none. However, both men and women are obtaining master's degrees; the disparity is rather small. It can be observed from the above data that men are more interested in obtaining various educational degrees. The table also indicates that the proportion of workers who complete their bachelor's degree before the age of 25 is high. Most master's degrees are completed or begun between the ages of 31 and 35. Furthermore, the proportion of workers who complete their bachelor's degree before the age of 25 is high. Most master's degrees are completed or begun between the ages of 31 and 35. For both the Ph.D. and the M.Phil., there is only one individual in the manufacturing business.

**Table 1:** Respondents Demographics.

Education Level	Age Group	Male	Female
O' levels/matric	Below 25	3	1
	26–30	0	2
	31–35	0	0
	36–40	0	0
	41–45	0	0
	Above 46	0	0
A' levels/intermediate	Below 25	13	6
	26–30	2	0
	31–35	0	0
	36–40	0	0
	41–45	0	0
	Above 46	0	0
Bachelor's degree	Below 25	51	46
	26–30	17	17
	31–35	3	0
	36–40	2	0
	41–45	0	0
	Above 46	1	1
Master's Degree	Below 25	36	21
	26–30	18	0
	31–35	25	1
	36–40	8	0
	41–45	1	0
	Above 46	1	0
M.Phil	Below 25	0	1
	26–30	0	0
	31–35	1	0
	36–40	0	0
	41–45	0	0
	Above 46	0	0
P.hd	Below 25	2	0
	26–30	0	0
	31–35	0	0
	36–40	1	0
	41–45	0	0
	Above 46	0	0

**Note:** the above table shows the cross tabulation between education and gender.

Table 2 shows the descriptive statistics where the values of the standard deviation are between 1 and 2, which shows that the values are normally distributed. However, if we look at each variable closely, the education values show a negative skewness, which shows that the value of skewness is less than 0.05 alpha and that there is no significant impact of education on this research topic. However, age shows that there is a positive skewness with a positive kurtosis, and this makes it easy to identify that age plays a significant role and is normally distributed in this table; hence, age has an effect on innovative behavior during employee engagement. However, gender has positive skewness and is larger than the alpha value of 0.05.

**Table 2:** Descriptive statistics.

	No.	Missing	Mean	Median	Min	Max	Standard Deviation	Excess Kurtosis	Skewness
Gender	1	0	1.417	1	1	2	0.493	−1.905	0.341
Education	2	0	3.211	3	1	6	0.767	1.728	−0.01
Age	3	0	1.794	1	1	6	1.068	1.742	1.384
EE1	5	0	3.839	4	1	5	1.017	−0.291	−0.629
EE2	6	0	3.894	4	1	5	1.057	−0.028	−0.784
EE3	7	0	3.928	4	1	5	1.121	−0.046	−0.86
EE4	8	0	3.506	4	1	5	1.209	−0.784	−0.384
EE5	9	0	3.294	3	1	5	1.272	−1.073	−0.16
EE6	10	0	3.728	4	1	5	1.115	−0.529	−0.561
EE7	11	0	3.606	4	1	5	1.003	−0.31	−0.277
IB1	12	0	3.639	4	1	5	0.953	−0.313	−0.306
IB2	13	0	3.711	4	1	5	1.019	−0.571	−0.379
IB3	14	0	3.572	4	1	5	1.106	−0.418	−0.408
IB4	15	0	3.589	4	1	5	1.063	−0.332	−0.514
EV1	16	0	3.511	4	1	5	1.077	−0.54	−0.325
EV2	17	0	3.433	3	1	5	1.096	−0.524	−0.212
EV3	18	0	3.711	4	1	5	1.019	−0.544	−0.411
EV4	19	0	3.583	4	1	5	1.09	−0.981	−0.151
EV5	20	0	3.756	4	1	5	0.981	−0.321	−0.453
PDF1	21	0	3.917	4	1	5	0.906	0.156	−0.648
PDF2	22	0	3.439	4	1	5	1.121	−0.445	−0.454
PDF3	23	0	3.467	4	1	5	1.103	−0.45	−0.379
PDF4	24	0	3.478	4	1	5	1.147	−0.44	−0.457
PDF5	25	0	3.606	4	1	5	1.171	−0.356	−0.591

**Note:** the above table shows that skewness and kurtosis of various question is within +3 and −3 which is an acceptable range.

Table 3 displays the correlation coefficients. Where values with ** show that the values are correlated at the level of 0.01.

**Table 3:** Correlation.

Correlations					
		EE	EVB	PDF	IWB
EE	Pearson Correlation	1	.386**	.199**	.287**
	Sig. (1-tailed)		.000	.007	.000
	N	180	180	180	180
EVB	Pearson Correlation	.386**	1	.020	.451**
	Sig. (1-tailed)	.000		.406	.000
	N	180	180	180	180
PDF	Pearson Correlation	.199**	.020	1	.201**
	Sig. (1-tailed)	.007	.406		.007
	N	180	180	180	180
IWB	Pearson Correlation	.287**	.451**	.201**	1
	Sig. (1-tailed)	.000	.000	.007	
	N	180	180	180	180
**. Correlation is significant at the 0.01 level (1-tailed).					

## 4 Results

### 4.1 Structural model

To analyze the reliability of all items, the researchers used the Cronbach's Alpha test, which is regarded acceptable if it surpasses a threshold of 0.70 [71]. The reliability data for employee engagement (EE), Employee voice behavior (EVB), perceived Distributive justice (PDJ), and innovative work behaviour (IWB) are provided in Table 4. All constructs values exceed the threshold of 0.70 (70 %) and therefore provide evidence to support the claim that the data can be regarded reliable. The range of loadings runs from 0.582 to 0.955. The average variance extracted (AVE), on the other hand, serve as markers of the convergent validity of our conceptions [72]. To be regarded adequate, the values of CR (composite reliability) should be greater than 0.7, and the average variance extracted (AVE) should be greater than 0.5. Table 4 shows that all CR values are greater than 0.7, and that all AVE values are greater than 0.5, both of which are considered acceptable levels of reliability. These findings show that the information gathered is trustworthy.

**Table 4:** Construct validity, convergent validity via AVE, and discriminant validity.

Construct	No. of items	Construct Reliability	Factor Loading Range	Average Variance Extracted (AVE)	EE	EVB	PDJ	IWB
EE	7	0.93	0.582–0.775	0.563				
EVB	5	0.82	0.521–0.725	0.572	0.467			
PDJ	5	0.89	0.612–0.955	0.522	0.412	0.467		
IWB	4	0.90	0.678–0.794	0.541	0.454	0.322	0.446	

**Note:** EE denotes employee engagement, EVB = Employee voice behavior, PDJ= Perceived distributive justice, and IWB= Innovative work behavior.

Furthermore, an HTMT value less than 0.85 is offered by Ref. [72] as an acceptable benchmark for measuring the discriminant validity of ideas. The results reported in Table 4 reveal that the HTMT values for all constructions were less than 0.85, indicating that the constructs are within an acceptable range.

The hypothesis is accepted and the link is considered significant when (p < 0.05). The relationship between employee engagement and innovative behaviour is statistically significant, as demonstrated in Table 5 (p = 0.026), which is less than 0.05, and thus supports the hypothesis. The link between employee engagement and employee voice is also statistically significant, as seen in the table, with a P value less than 0.05. The correlation between employee voice and innovative behavior is statistically significant, as evidenced by a P value of less than 0.05.

**Table 5:** Direct relationship.

	Beta	Standard Error	T Statistics	P Values	Findings
H1: EE - > IB	0.117	0.06	1.937	0.026	Supported
H2a: EE - > EV	0.491	0.061	8.015	0.000	Supported
H2b: EV - > IB	0.631	0.063	10.065	0.001	Supported

**Note:** the above relationships are supported.

In Table 6, this study demonstrates how employee voice can act as a mediator between employee engagement and innovative behaviour. The findings presented in Table 6 of this research study reveal a p-value of 0, or p < 0.05, which signifies that there is a statistically significant relationship between employee engagement and innovative behaviour. This outcome supports the notion of an indirect correlation between the variables under investigation. According to Table 6, the mediation analysis results indicate that the original sample has a value of 0.31, the T statistic is 6.078, and the P value is 0.

**Table 6:** Indirect relationship (mediation analysis).

	Beta	Standard Error	T Statistics	P Values	Findings
H2: EE - > EV - > IB	0.31	0.051	6.078	0	Supported

**Note:** the above relationship is supported.

The primary objective of a moderator analysis is to ascertain the extent to which the importance of a third variable influences or alters the relationship between two variables. In the aforementioned Table 7, the p-value of the variable exceeds the significance level of 0.05, commonly referred to as alpha, specifically amounting to 0.08. Based on statistical analysis, the relationship between employee engagement and innovative work behaviour does not appear to be influenced by perceived distributive fairness, hence lacking support.

**Table 7:** Moderating analysis.

	Beta	Standard Error	T Statistics	P Values	Findings
H3: PDF*EE - > IB	0.081	0.059	1.37	0.085	Not supported

**Note:** the above relationship is not supported.

## 5 Discussion

The objective of this study was to explore an area that has been minimally addressed in the existing body of literature. The objective of this study was to examine and comprehend the direct and indirect effects of employee engagement on employees' innovative work behaviour, with the mediating role of employee voice behavior. The investigation also examined the moderating relationship between perceived distributive justice and the relationship between EE and IWB.

The study findings revealed the positive effect of employee engagement on innovative work behavior, showing that its p-value is less than 0.05, indicating that employee engagement has a statistically significant positive influence on innovative work behaviour. This demonstrates that our H1 is being supported. The H1 findings are also supported by relevant studies, for example, those reconfirmed by Ref. [73]. In the literature, innovation has been related to other factors, or the linkage between innovative behavior and employee engagement has been disputed. The research focuses on the relationship between employee engagement and innovative behaviour. Additionally, prior research has shown that employee engagement is an attitude component that promotes innovative behaviour inside the firm. According to Ref. [46], any amount of positive involvement at work influences innovative behaviour. Motivated workers show proof that engagement and innovative activity are strongly associated [74,48,75]. [76] concur that employee engagement is a crucial factor in fostering innovative behaviour [77]. argued that the greater the level of employee engagement, the greater the likelihood of innovative uniqueness. Employee behavior was a significant influence on the success of any firm, and individuals with more autonomy were more innovative and engaged in their work [55]. According to Ref. [78], employee engagement provides a strong motivating foundation for a desired action, and it is considered that engaged people perform more efficiently [17]. According to Ref. [79], employee engagement is the driving force behind innovative behaviour. Engaged workers like emulating ways of overcoming obstacles and acting creatively.

Moreover, scholarly research suggests that employees who are actively involved in their work demonstrate higher levels of innovation. The observation that employee voice serves as a mediator between employee engagement and innovative behaviour highlights a positive and indirect association between employee engagement and innovative behaviour [80]. The results of the study indicate that there is an indirect relationship between employee engagement and workers' innovative behaviour. This relationship is mediated by employee voice, and it ultimately leads to an enhancement in organizational credibility. This conclusion aligns with the second objective of our study. The finding that the mediating hypothesis yields a p-value below 0.05 indicates that there is evidence to suggest that employee voice has a beneficial impact on innovative behaviour. This result confirms the existence of an indirect relationship between the variables under investigation and provides support for the mediating hypothesis proposed in our study. Employee voice and employee engagement improve innovative behavior, as evidenced in the study, where Employees' innovative behavior, results in an innovative company, and an innovative organization fosters the culture and trend of employee voice. The results confirmed the full-mediating effect [81]. Employee voice not only increases workers' confidence, representation, and morale [33], but it also enhances organizational performance with the company's projected innovative capabilities [30] employee voice, innovative behavior, and engagement because confident employees appear to have a greater degree of confidence in management, and confidence in the organization is related to higher levels of employee engagement.

Prior research attempted to explain the relationship between employee engagement and innovative behavior, but because employees' difficulties and skills change over time, we employed perceived distributive fairness as a moderator of this relationship. However, despite the findings indicating innovative behavior, there is a general lack of support for perceived distributive fairness for employee engagement as the p-value is greater than 0.05, and thus, we are unable to ascertain the values that support this assertion. This demonstrates that our moderating hypothesis is not supported. The data and results imply that the relationship between perceived distributive justice and employee engagement is independent of creative activity; however, because The importance of perceived distributive justice is overstated in the literature. Employers, according to Ref. [82], are less likely to accept new ideas proposed by innovative staff, which leads to conflicts. Yet, perceived distributive fairness works as a mediating variable between innovative behavior and employee engagement, lowering the incidence of employee conflicts but having no substantial positive influence on employee engagement or innovative behavior. perceived distributive justice are more likely to leave the company because their energizing routines and innovative perspectives help them find a better position. Perceived distributive fairness supports individual employee innovation but not team innovation [30], and it frequently has a detrimental effect on workers' engaged behavior [30,83]. Previous studies, such as [84,85,86], indicated that distributive fairness can affect both positive and negative attitudes in the workplace and that it primarily has a favorable effect on job satisfaction, salary, and commitment [87,88]. According to Ref. [40], workers who are not highly engaged may face stress as a result of distributive justice. It has been further argued that while perceived distributive justice addresses the fairness of rewards and outcomes, it has no direct effect on an individual's propensity to generate innovative ideas or engage in innovation-related activities [89]. The moderating effect of distributive justice may be more pronounced in contexts explicitly related to remuneration or equitable resource allocation, whereas the promotion of innovative behaviours frequently requires a broader variety of organizational factors, such as supportive leadership [90].

### 5.1 Practical implications

The findings of this research indicate that employee engagement promotes employee voice, which in turn leads to innovative workplace behavior. The manufacturing business has a basic requirement for workers that exhibit innovative behaviour, and in order to meet this demand, organisations must comprehend the relevance of employee engagement and employee voice, through which employees may contribute to the company's efficient operation. Employees that are allowed autonomy (employee voice) outperform those who keep their ideas to themselves when it comes to innovative behavior, according to our research. Employee engagement also has a direct association with innovative behaviour, according to our results. With changing market dynamics and increased competition among firms, innovative behaviour is becoming a core need for all organisations, and corporations must increasingly devise strategies to promote innovative behaviour in the workplace. When managers provide their workers with work freedom and the chance to provide input on any task or assignment, people feel committed to their jobs and exhibit innovative behaviour. Managers of separate departments contribute significantly to the productivity of their teams and the department as a whole; via their participation, they may stimulate innovative behaviour among their workers. To incorporate employee voice into the organization's culture, it is essential to value their opinion, which can be lost due to the fear of failure; therefore, employees should be reassured that making mistakes is a part of the learning curve and managers should talk them through whenever employees seem to fall through the cracks. No matter how dedicated an employee is to the job, work can become overwhelming at times; however, through open communication and a little patience, managers can help employees overcome these obstacles. In such an atmosphere, employees will not feel compelled to keep their opinions to themselves and will be more comfortable discussing their disruptive behaviour.

In sum, the study provides some important insights for the Pakistani manufacturing industry. A company retains control over its innovation as a result of the substantial participation of its staff. The manufacturing sector is vital to the economy since it creates income and provides economic support. Innovation is essential to the proper operation and long-term survival of industrial enterprises. Rapid globalization has resulted in increased competitiveness; thus, Pakistan's manufacturing industry must create and implement innovation, not only in terms of new products but also innovative staff behavior. To compete worldwide and domestically, the manufacturing industry requires creativity and innovation. Creativity is the generation of ideas, whereas innovative behavior is their application. Innovative behavior is a word used in human resources. Employees contribute their innovative behaviour by expressing fresh and original ideas and putting those ideas into practice in order to create innovative goods. For instance, an employee may recommend adopting a new technology that produces more things in less time. Another instance is when staff recommend purchasing high-grade raw materials to increase quality. Innovative behaviour emanates from workers, whose contributions to the company are processed by equipment to provide an innovative final result. Innovative behaviour is a valuable asset for manufacturing businesses because it incorporates new and original ideas for work, goods, and services, enhancing the performance of people, departments, and the company as a whole. It may be used to deal with the complicated environment of the industrial industry. In the current age of globalization, it is a crucial aspect of maintaining competitiveness, and as such, it may push the industrial sector toward greater efficiency. Employee involvement, flexibility, and employee voice serve to stimulate innovative behaviour. Engaged workers who are engaged in decision and policy making are encouraged to communicate their opinions proactively, which fosters creative behaviour. The manufacturing industry is implementing workplace flexibility to promote employee engagement so that workers feel happy and share their voice (create new ideas), which results in creative behaviour and so enhances the firm's overall efficiency.

### 5.2 Limitations

Although there were several limitations to this study, they can serve as a starting point for additional research. The sample for this research study was made up of employees especially working in Pakistan's manufacturing industry. Hence, it would be intriguing to explore an alternative geographic region for future study endeavours. Another limitation of this study was its reliance on cross-sectional data, which may have limited the causal effect of employee engagement and innovative work behaviour. It is recommended that future research endeavours employ longitudinal data in order to ascertain the definitive impact of interpersonal interactions.

### 5.3 Future research direction

Future research on the impact of employee engagement on employees’ innovative behavior through the mediation of employee voice and moderation of perceived distributive fairness should address the limitations of this study. One avenue for future research is to explore the moderating effect of perceived distributive justice more in-depth and to determine if it has an impact on the relationship between employee engagement and innovative behavior. Another direction for future research is to expand the scope of the study beyond the manufacturing sector and examine the impact of employee engagement on innovative behavior in different industries. Additionally, future research should consider other factors, such as workplace flexibility, that may impact employee engagement and innovative behavior. Furthermore, future studies may also consider other dependent variables besides innovative behavior, such as the performance of the firm, to fully understand the impact of employee engagement on the company. In conclusion, this study highlights the importance of employee voice as a mediator in the relationship between employee engagement and innovative behavior, but there is still much to be explored in this field.

This study's findings illustrate the importance of employee voice as a mediator between employee engagement and innovative Behavior. Thus, employee egagement generates employee voice, which in turn generates innovative behaviour. In this research, however, the moderating effect of perceived distributive justice has not been shown. Future researchers will be tasked with addressing the study's weaknesses. First, the PLS- SEM results do not support the moderating impact of perceived distributive justice, paving the way for further study. Second, the respondent sample is restricted to manufacturing sector personnel. Future researchers should behavior further industry-specific studies to improve the generalizability of this work. Additionally, other factors, such as workplace flexibility, should be studied. This study's final dependent variable was innovative behavior, although for the evaluation of a firm's performance, other factors might be examined. Further research should examine the links between numerous independent and dependent factors that may influence employee engagement and innovative behaviour.

## Data Availability Statement

Because of the nature of this study, participants did not give permission for their data to be shared publicly; hence, supporting data are unavailable.

## CRediT authorship contribution statement

**Bilqees Ghani:** Writing – original draft. **Syed Irfan Hyder:** Investigation, Conceptualization. **Sunghoon Yoo:** Writing – review & editing, Funding acquisition. **Heesup Han:** Writing – review & editing, Project administration, Funding acquisition.

## Declaration of competing interest

The authors declare no conflict of interest.
